# Factors associated with burnout among frontline nurses in the post-COVID-19 epidemic era: a multicenter cross-sectional study

**DOI:** 10.1186/s12889-024-18223-4

**Published:** 2024-03-04

**Authors:** Shitao Wang, Guoshuai Luo, XiangQian Ding, Xuelu Ma, Fei Yang, Mengen Zhang, Guangxin Sun, Fei Wang, Liping Zhu, Shuo Wang, Zongyou Li

**Affiliations:** 1 Department of Neurology, Affiliated Fuyang People’s Hospital of Anhui Medical University, Fuyang, China; 2 Laboratory of Biological Psychiatry, Institute of Mental Health, Tianjin Anding Hospital, Mental Health Center of Tianjin Medical University, Tianjin, China; 3 Department of Neurosurgery, Qilu Hospital of Shandong University, Jinan, China; 4 Department of Neurology, Affiliated Hospital of North Sichuan Medical College, Nanchong, China; 5 Ya’an People’s Hospital, Sichuan University, Yaan, China

**Keywords:** Burnout, COVID-19, Nurse, Post-pandemic, Prevalence

## Abstract

**Background:**

The COVID-19 pandemic has significantly increased the risk of burnout among frontline nurses. However, the prevalence of burnout and its associated factors in the post-pandemic era remain unclear. This research aims to investigate burnout prevalence among frontline nurses in the post-pandemic period and pinpoint associated determinants in China.

**Methods:**

From April to July 2023, a cross-sectional study was carried out across multiple centers, focusing on frontline nurses who had been actively involved in the COVID-19 pandemic. The data collection was done via an online platform. The Maslach Burnout Inventory-Human Services Survey was utilized to evaluate symptoms of burnout. A multivariable logistic regression analysis was used to pinpoint factors associated with burnout.

**Results:**

Of the 2210 frontline nurses who participated, 75.38% scored over the cut-off for burnout. Multivariable logistic regression revealed that factors like being female [odds ratio (OR) = 0.41, 95%CI = 0.29–0.58] and exercising 1–2 times weekly[OR = 0.53, 95%CI = 0.42–0.67] were protective factors against burnout. Conversely, having 10 or more night shifts per month[OR = 1.99, 95%CI = 1.39–2.84], holding a master’s degree or higher[OR = 2.86, 95% CI = 1.59–5.15], poor health status[OR = 2.43, 95% CI = 1.93–3.08] and [OR = 2.82, 95%CI = 1.80–4.43], under virus infection[OR = 7.12, 95%CI = 2.10-24.17], and elevated work-related stress[OR = 1.53, 95% CI = 1.17-2.00] were all associated with an elevated risk of burnout.

**Conclusion:**

Our findings indicate that post-pandemic burnout among frontline nurses is influenced by several factors, including gender, monthly night shift frequency, academic qualifications, weekly exercise frequency, health condition, and viral infection history. These insights can inform interventions aimed at safeguarding the mental well-being of frontline nurses in the post-pandemic period.

## Introduction

Nurse burnout is a well-documented work-related stress syndrome characterized by emotional exhaustion, depersonalization, and reduced personal accomplishment [1]. Research from 2012 indicated a heightened prevalence of burnout among frontline nurses [2], which is consistent with the results of a study conducted in 2018 [3]. Various factors, including exposure to violence [4], excessive workload [5], Post-Traumatic Stress Disorder [6] and insomnia [7], contribute to this phenomenon. The implications of nurse burnout are profound, potentially leading to a decline in the quality of patient care [8]. The emergence of the COVID-19 pandemic further exacerbated the issue of nurse burnout [9, 10]. During the pandemic, elevated stress levels and exposure to traumatic events were notably correlated with an increased risk of burnout in frontline nurses [11–14]. In addition, poor staffing ratios are a significant concern, with research indicating that a nurse-to-patient ratio exceeding 1:2 amplifies the risk of burnout for nurses in intensive care units [15, 16]. The epidemic has led to a surge in burnout among frontline nurses, thereby increasing the probability of unfavorable nursing incidents.

Although factors contributing to nurse burnout were extensively studied during the pandemic, the post-epidemic prevalence and determinants of burnout among frontline nurses remain unclear. This study aims to ascertain the prevalence and underlying causes of burnout among frontline nurses in China in the aftermath of the COVID-19 pandemic.

## Methods

### Study design

A cross-sectional study was conducted between April and July 2023, subsequent to the COVID-19 pandemic in China. The study population comprised frontline nurses holding valid professional qualification certificates. Descriptive characteristics of the nurses were gathered via the Wenjuanxing platform (https://www.wjx.cn). Initially, we signed up for the Wenjuanxing platform and subsequently imported the questionnaire content into it. This process enabled us to obtain a link to the questionnaire. We then disseminated this link to the nurses’ mobile phones through WeChat (a widely-used social application in China with over 1 billion active users), facilitating timely completion of the survey.

### Instruments and measures

The following measures and questions were collected:

1. Descriptive characteristics of nurses: This included job title, gender, employment status, monthly frequency of night shifts, qualification, age, weekly frequency of exercise, personality traits, health status, history of virus infection, economic pressures, lifestyle, work-related stress, and concerns regarding potential infection. Considering that these descriptive characteristics may be related to the burnout of frontline nurses, and these specific responses can more accurately reveal the relationship between these descriptive characteristics and the burnout of frontline nurses, they were selected for this survey. Burnout Assessment: Burnout was evaluated using the Maslach Burnout.
2. Inventory-Human Services Survey (MBI-HSS), a validated instrument for assessing burnout among healthcare professionals [17–19]. This tool has demonstrated correlations with the quality of care [20]. Comprising 22 items, respondents rate each on a 7-point scale, from 0 (Never) to 6 (Every day). The scale evaluates three domains: emotional exhaustion, depersonalization, and reduced personal achievement. Cut-off scores of > 26, >9, and < 33 are indicative of clinically significant emotional exhaustion, depersonalization, and reduced personal achievement, respectively [21]. Being at high risk of burnout in at least one of the three domains is deemed as experiencing burnout [22]. The Cronbach’s α for the Chinese version of the MBI-HSS stood at 0.830 [23], signifying a substantial degree of internal consistency.

### Statistical analyses

Statistical analyses were conducted using IBM SPSS Statistics 23.0 and GraphPad Prism 9.0 software. Frequency distributions were treated as categorical variables and compared between groups using the chi-square test. To adjust for multiple testing, the Bonferroni correction was applied, with a *p*-value < 0.004 (0.05/14) deemed statistically significant. Multivariate regression analyses were employed to examine the relationship between nurses’ descriptive characteristics and burnout, setting the significance threshold at *p* < 0.004 (0.05/14). Variables selected for the adjusted analysis encompass job title, gender, employment status, monthly frequency of night shifts, qualification, age, weekly frequency of exercise, personality trait, health status, virus infection, economic pressure, lifestyle, work pressure, and concern about potential infection.

## Results

### Description of nurse characteristics

A total of 2,210 nurses from 27 provinces across China participated in the survey. Of these, 41.31% held the position of nurse-in-charge, and a significant majority, 80.27%, were female. Permanent employment was reported by 31.04% of the respondents, while 45.02% undertook between 5 and 10 night shifts monthly. The predominant age bracket was 25 to 36 years, encompassing 51.99% of the participants, and 66.20% held an undergraduate degree with with a specialisation. More nurses’ characteristics are provided in Table 1. The distribution of risk factors related to nurse burnout across the entire sample is detailed in Table 2.

**Table 1:** Nurses’ descriptive characteristics

Descriptive Characteristic		*N*	%	
Job title				
	Nurse (and below)	523	23.67	
	Nurse practitioner	774	35.02	
	Nurse-in-charge (and above)	913	41.31	
Gender				
	Male	436	19.73	
	Female	1774	80.27	
Employment status				
	Permanent employment	686	31.04	
	Temporary employment	1524	68.96	
Frequency of night shifts per month				
	<5	830	37.56	
	5 ∼ 10	995	45.02	
	>10	385	17.42	
Qualification				
	Specialty (and below)	516	23.35	
	Undergraduate college	1463	66.20	
	Master (and above)	231	10.45	
Age				
	≤ 25	379	17.15	
	26 ∼ 35	1149	51.99	
	36 ∼ 45	488	22.08	
	> 45	194	8.78	
Frequency of exercise per week				
	Never	1002	45.34	
	1–2	800	36.20	
	3(and above)	408	18.46	
Personality trait				
	Introvert	1159	52.44	
	Extroversion	1051	47.56	
Health status				
	Good	1048	47.42	
	General	909	41.13	
	Chronic disease	253	11.45	
Virus infection				
	Under infection	134	6.06	
	Recovery	1863	84.29	
	No infection	213	9.64	
Economic pressure				
	Yes	1614	73.03	
	No	596	26.97	
Living style				
	Living alone	424	19.19	
	Living with family	1506	68.14	
	Living with colleagues	280	12.67	
Working pressure				
	Yes	1632	73.85	
	No	578	26.15	
Concern about potential infection				
	Yes	1116	50.50	
	No	1094	49.50	

**Table 2:** Distribution of risk factors across nurse burnout in the overall sample(emotional exhaustion > 26, depersonalisation > 9, reduced personal achievement < 33)

Descriptive Characteristic		Job burnout		χ ^2^	*P*
		Yes	No		
Job title				6.156	0.046
	Nurse (and below)	395(17.87%)	128(5.79%)		
	Nurse practitioner	605(27.38%)	169(7.65%)		
	Nurse-in-charge (and above)	666(30.14%)	247(11.18%)		
Gender				45.439	<0.001
	Male	383(17.33%)	53(2.40%)		
	Female	1283(58.05%)	491(22.22%)		
Employment status				5.954	0.015
	Permanent employment	540(24.43%)	146(6.61%)		
	Temporary employment	1126(50.95%)	398(18.01%)		
Frequency of night shifts per month				52.283	<0.001
	<5	565(25.57%)	265(11.99%)		
	5 ∼ 10	767(34.71%)	228(10.32%)		
	>10	334(15.11%)	51(2.31%)		
Qualification				47.909	<0.001
	Specialty(and below)	396(17.92%)	120(5.43%)		
	Undergraduate college	1055(47.74%)	408(18.46%)		
	Master (and above)	215(9.73%)	16(0.72%)		
Age				7.938	0.047
	≤ 25	300(13.57%)	79(3.57%)		
	26 ∼ 35	873(39.50%)	276(12.49%)		
	36 ∼ 45	347(15.70%)	141(6.38%)		
	> 45	146(6.61%)	48(2.17%)		
Frequency of exercise per week				49.071	<0.001
	Never	801(36.24%)	201(9.10%)		
	1–2	535(24.21%)	265(11.99%)		
	3(and above)	330(14.93%)	78(3.53%)		
Personality trait				4.859	0.028
	Introvert	896(40.54%)	263(11.90%)		
	Extroversion	770(34.84%)	281(12.71%)		
Health status				113.998	<0.001
	Good	684(30.95%)	364(16.47%)		
	General	756(34.21%)	153(6.92%)		
	Chronic disease	226(10.23%)	27(1.22%)		
Virus infection				46.174	<0.001
	Under infection	131(5.93%)	3(0.14%)		
	Recovery	1361(61.58%)	502(22.71%)		
	No infection	174(7.87%)	39(1.76%)		
Economic pressure				5.613	0.018
	Yes	1238(56.02%)	376(17.01%)		
	No	428(19.37%)	168(7.60%)		
Living style				35.980	<0.001
	Living alone	357(16.15%)	67(3.03%)		
	Living with family	1079(48.82%)	427(19.32%)		
	Living with colleagues	230(10.41%)	50(2.26%)		
Working pressure				12.706	<0.001
	Yes	1262(57.10%)	370(16.74%)		
	No	404(18.28%)	174(7.87%)		
Concern about potential infection				13.566	<0.001
	Yes	804(36.38%)	312(14.12%)		
	No	862(39.00%)	232(10.50%)		

### Burnout prevalence and associated risk factors

**Fig. 1:**
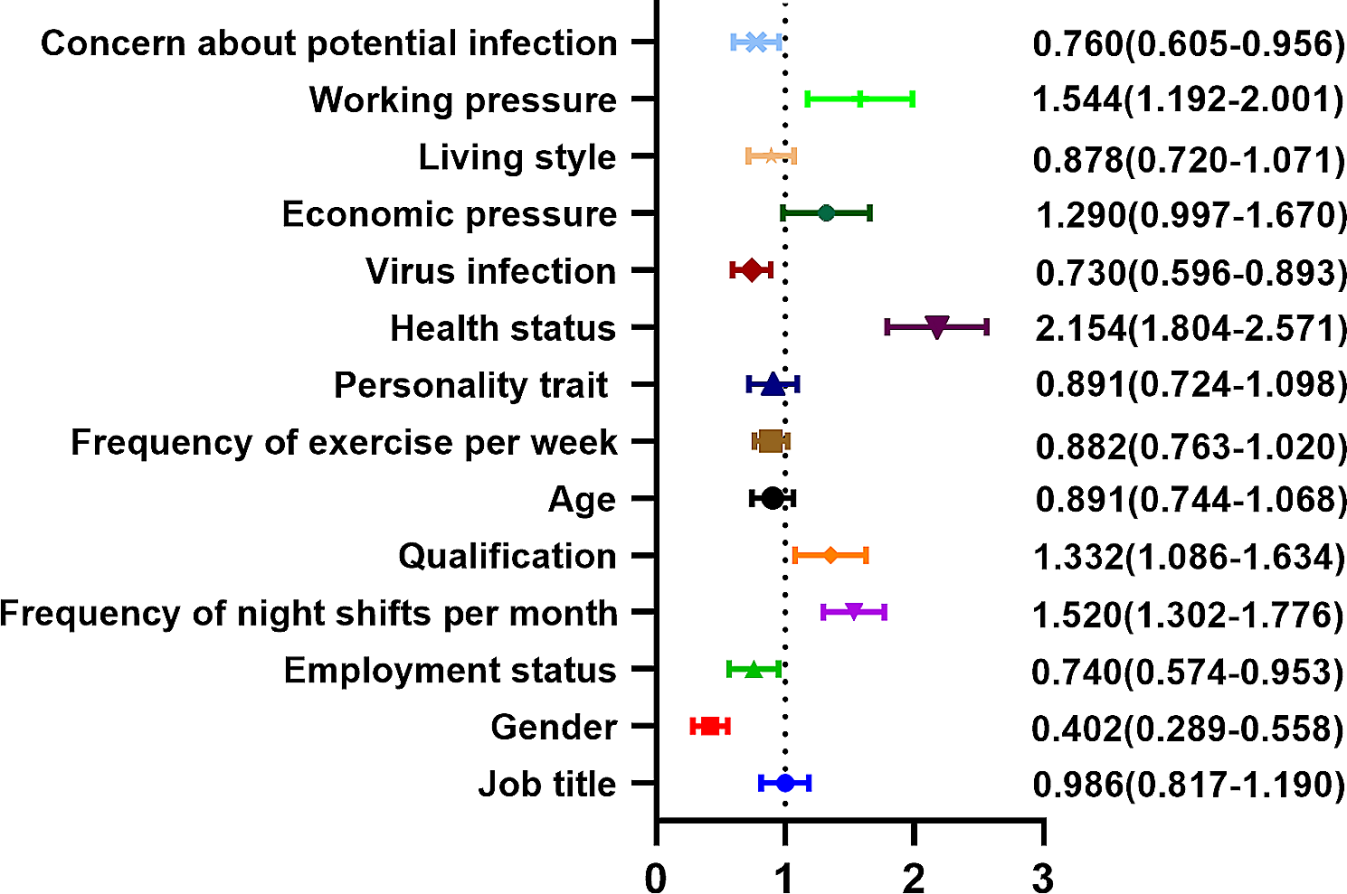
Forest plot for logistic regression analysis of the factors of burnout among frontline nurses

The prevalence of burnout among frontline nurses in this study was 75.38% (1,666 out of 2,210). The regression analysis concerning nurses’ descriptive characteristics is illustrated in Fig. 1. After adjusting for multiple testing (as seen in Table 3), factors like being female and exercising 1–2 times weekly were found to be protective against burnout. Conversely, having five or more night shifts monthly, holding a master’s degree or higher, poor health status, under virus infection, and elevated work-related stress were all associated with an elevated risk of burnout.

**Table 3:** Factors associated with burnout risk(emotional exhaustion > 26, depersonalisation > 9, reduced personal achievement < 33 )

Descriptive Characteristic	Adjusted OR (95% CI)	Category *p* value	Overall *p* value
**Gender**			
Male	1		<0.001
Female	0.412(0.294–0.579)^a^	0.000	
**Frequency of night shifts per month**			
<5	1		<0.001
5 ∼ 10	1.410 (1.120–1.776)	0.004	
>10	1.985 (1.389–2.837)^b^	0.000	
**Qualification**			
Specialty (and below)	1		<0.001
Undergraduate college	0.858(0.643–1.146)	0.300	
Master (and above)	2.860 (1.587–5.153)^c^	0.000	
**Frequency of exercise per week**			
Never	1		<0.001
1–2	0.528(0.417–0.668)^d^	0.000	
3(and above)	0.981(0.705–1.364)	0.907	
**Health status**			
Good	1		<0.001
General	2.434(1.926–3.075)^e^	0.000	
Chronic disease	2.819(1.796–4.426)^f^	0.000	
**Virus infection**			
No infection	1		0.001
Under infection	7.120(2.097–24.170)^g^	0.002	
Recovery	0.775(0.518–1.161)	0.216	
**Working pressure**			
No	1		0.002
Yes	1.530(1.169–2.002)^h^	0.002	

^a^OR: Adjustment for job title, employment status, frequency of night shifts per month, qualification, age, frequency of exercise per week, personality trait, health status, virus infection, economic pressure, living style, working pressure and concern about potential infection; ^b^OR: Adjustment for job title, gender, employment status, qualification, age, frequency of exercise per week, personality trait, health status, virus infection, economic pressure, living style, working pressure and concern about potential infection; ^c^OR: Adjustment for job title, gender, employment status, frequency of night shifts per month, age, frequency of exercise per week, personality trait, health status, virus infection, economic pressure, living style, working pressure and concern about potential infection; ^d^OR: Adjustment for job title, gender, employment status, frequency of night shifts per month, qualification, age, personality trait, health status, virus infection, economic pressure, living style, working pressure and concern about potential infection; ^e^OR,^f^OR: Adjustment for job title, gender, employment status, frequency of night shifts per month, qualification, age, frequency of exercise per week, personality trait, virus infection, economic pressure, living style, working pressure and concern about potential infection; ^g^OR: Adjustment for job title, gender, employment status, frequency of night shifts per month, qualification, age, frequency of exercise per week, personality trait, health status, economic pressure, living style, working pressure and concern about potential infection; ^h^OR: Adjustment for job title, gender, employment status, frequency of night shifts per month, qualification, age, frequency of exercise per week, personality trait, health status, virus infection, economic pressure, living style and concern about potential infection

## Discussion

This study evaluated burnout and its associative factors among frontline nurses after the COVID-19 pandemic. Our findings pinpointed several determinants linked to burnout in frontline nurses, including gender, monthly frequency of night shifts, qualification, weekly exercise frequency, health status, and history of viral infection. Along with our study, an increasing body of research has pinpointed factors that affect the risk of burnout among nurses in the post-pandemic era. These studies can offer valuable insights for interventions aimed at mitigating nurse burnout after the pandemic [24].

Regular exercise can effectively curb the incidence of occupational burnout among oncologists [25]. Our research indicates that after the COVID-19 pandemic, 75.38% of nurses experienced burnout symptoms, encompassing emotional exhaustion, depersonalization, and reduced personal achievement. These results is consistent with a survey undertaken in China during the pandemic [12] but are notably higher than findings from other countries [26–29]. Several factors might account for this discrepancy: Primarily, variations in the work environment and the specific phase of the pandemic play pivotal roles in these divergent outcomes. Additionally, some studies that exclusively gauge burnout by assessing emotional exhaustion tend to report a lower prevalence. Lastly, the use of different assessment instruments can also introduce variability in results. Moreover, the readiness of health systems, potential understaffing in health organisations, workload, and other organisational factors also significantly contribute to this discrepancy.

Our research indicates that gender plays a role in burnout among frontline nurses, with females showing a lower prevalence compared to males. This observation aligns with certain previous studies [30, 31]. Another significant factor associated with burnout identified in this study is the frequency of night shifts per month. Specifically, nurses working more than 10 night shifts monthly are at a considerably heightened risk of burnout. Understaffing could be the primary cause for the increased frequency of night shifts observed among certain nurses. This correlation between the number of night shifts and elevated MBI scores is supported by earlier findings [32, 33]. Furthermore, our study discerned a link between burnout and educational qualifications. Interestingly, nurses possessing graduate degrees appear more susceptible to burnout, a trend previously observed among medical educators [34]. The primary reason that nurses with advanced educational qualifications are more susceptible to burnout is due to their excessive workload, coupled with the additional responsibility of conducting scientific research, a requirement not typically imposed on nurses with lower education levels.

Our research indicates that engaging in moderate exercise (once to twice a week) post-epidemic can considerably reduce burnout risk among frontline nurses, corroborating the outcomes of a recent study [35]. Intriguingly, we did not identify a direct correlation between extremely high or low exercise frequencies and burnout prevalence. While several reports highlight a strong relationship between poor health status and burnout [36, 37], our findings align with these, though another study detected no impact of health status on the Maslach Burnout total score [38]. The discrepancy across studies might stem from geographical differences in research areas. Notably, our comprehensive survey spanned 27 provinces and exclusively focused on frontline nurses, unlike other studies. In our study, a nurse’s viral infection status emerged as a critical factor linked to burnout. Understandably, frontline nurses infected with the virus often grapple with compromised health, amplifying their burnout risk. This aligns with our earlier observation regarding the association between poor health and increased burnout risk. Furthermore, job-related stress was identified as a burnout risk, echoing another study’s findings [39]. Interestingly, a prior study demonstrated that health-related quality of life, another measure of personal health, exhibited a strong correlation with burnout [40]. Nevertheless, further investigations are essential to validate these insights.

Although our multicenter study rigorously assessed the associations between various factors and post-pandemic burnout among frontline nurses, there are several limitations to consider: (1) Our research focused solely on China, potentially not capturing the unique experiences and mental health trajectories of frontline nurses in other cultural or national contexts; (2) Although we endeavored to encompass a diverse sample across multiple provinces, disparities in healthcare settings, the pandemic’s impact, and socioeconomic nuances across these regions might impede the wider applicability of our findings; (3) Even though we adjusted for numerous demographic elements, potential unaccounted confounders might still sway the identified correlations between burnout and the variables examined; (4) In certain provinces, the sample sizes were comparatively limited, which could potentially introduce bias into the outcomes; (5) Some descriptive characteristics of nurses (weekly frequency of exercise, personality traits, health status) were self-reported.

## Conclusion

Our research reveals a higher prevalence of burnout among frontline nurses in the post-COVID-19 epidemic era. We identified several influencing factors, including gender, monthly night shift frequency, educational qualification, weekly exercise frequency, health status, and viral infection status. These insights are invaluable for strategizing interventions to manage and alleviate burnout among frontline nurses in the aftermath of the COVID-19 pandemic.

## Data Availability

The raw data supporting the results of this study are available from the corresponding author upon reasonable request.

## Abbreviations

COVID-19: Coronavirus disease
MBI-HSS: Maslach Burnout Inventory-Human Services Survey
